# Subclinical Mastitis in Lacaune Sheep: Etiologic Agents, the Effect on Milk Characteristics, and an Evaluation of Infrared Thermography and the YOLO Algorithm as a Preprocessing Tool for Advanced Analysis

**DOI:** 10.3390/vetsci11120676

**Published:** 2024-12-22

**Authors:** Marios Lysitsas, Georgios Botsoglou, Dimitris Dimitriadis, Sofia Termatzidou, Panagiota Kazana, Grigorios Tsoumakas, Constantina N. Tsokana, Eleni Malissiova, Vassiliki Spyrou, Charalambos Billinis, George Valiakos

**Affiliations:** 1 Faculty of Veterinary Science, University of Thessaly, 43100 Karditsa, Greece; mlysitsas@uth.gr (M.L.); billinis@uth.gr (C.B.); 2 School of Informatics, Aristotle University of Thessaloniki, 54124 Thessaloniki, Greece; gbotso@csd.auth.gr (G.B.); dndimitri@csd.auth.gr (D.D.); greg@csd.auth.gr (G.T.); 3 Department of Animal Science, University of Thessaly, 41334 Larissa, Greece; stermatzidou@uth.gr (S.T.); malissiova@uth.gr (E.M.); vasilikispyrou@uth.gr (V.S.); 4 Department of Agriculture, University of Ioannina, 47100 Arta, Greece; pankazana@philosophy.uoa.gr; 5 School of Veterinary Medicine, Faculty of Health Sciences, Aristotle University of Thessaloniki, 54124 Thessaloniki, Greece; ctsokana@vet.auth.gr

**Keywords:** antimicrobial resistance, coagulase-negative, infrared thermography, Lacaune, somatic cell count, staphylococci, subclinical mastitis, thermal images, YOLO algorithm

## Abstract

This study aimed to investigate the incidence of subclinical mastitis (SCM), the implicated pathogens, and their impact on milk quality in dairy sheep in Greece. Furthermore, we preliminarily evaluated infrared thermography and the application of AI tools for the early, non-invasive diagnosis of relevant cases. In total, 660 milk samples and over 2000 infrared thermography images were obtained from 330 phenotypically healthy ewes. Microbiological investigations, a somatic cell count (SCC), and milk chemical analyses were performed. Infrared images were analyzed using the FLIR Research Studio software (version 3.0.1). The You Only Look Once version 8 (YOLOv8) algorithm was employed for the automatic detection of the udder’s region of interest. A total of 157 mammary glands with SCM were identified in 122/330 ewes (37.0%). The most prevalent pathogen was staphylococci (136/160, 86.6%). Considerable resistance was detected to tetracycline (29.7%), ampicillin (28.6%), and sulfamethoxazole–trimethoprim (23.6%). SCM correlated with high total mesophilic count (TMC) values and decreased milk fat, lactose, and protein content. A statistically significant variation (*p* < 0.001) was identified in the unilateral SCM cases by evaluating the mean temperatures of the udder region between the teats in the thermal images. Finally, the YOLOv8 algorithm was employed for the automatic detection of the udder’s region of interest (ROI), achieving 84% accuracy in defining the ROI in this preliminary evaluation. This demonstrates the potential of infrared thermography combined with AI tools for the diagnosis of ovine SCM. Nonetheless, more extensive sampling is essential to optimize this diagnostic approach.

## Simple Summary

Subclinical mastitis (SCM) is a troublesome disease in dairy sheep that is highly prevalent among lactating ewes and regularly underdiagnosed. This study aimed to identify and investigate relevant SCM cases in Lacaune sheep in Greece and evaluate infrared thermography as an auxiliary tool for the immediate and easy detection of SCM cases at the farm level. The results highlighted the high prevalence of SCM in the examined farms, the predominance of staphylococci as etiologic agents, and the negative effect on milk quality indicators, such as somatic cells and the chemical composition. Moreover, the antibiotic resistance rates of the implicated bacteria against some widely used agents are undoubtedly concerning. The analysis of thermal images using the relevant software revealed specific variations in the mean temperatures in a specific udder region, which could be evaluated for the classification of the examined animals. Additionally, an artificial intelligence (AI) object detection method was employed for the automatic detection of the teats, indicating that this technology could be utilized for the detection of the region of interest (ROI) in infrared photos. The results highlight the potential of infrared technology and AI for the creation of a tool that could be utilized in the diagnosis of ovine subclinical mastitis in the future. However, there is a need for more extensive sampling and investigation in the future to rationalize the technique and provide a new addition to the armory of veterinarians.

## 1. Introduction

Mastitis is a pathological condition of great importance in sheep flocks, especially in lactating breeds [1,2]. Although clinical mastitis is acute, more severe, and requires treatment, the majority of cases encountered in sheep belong to the subclinical type of disease [3]. Subclinical mastitis (SCM) is a cause of significant economic loss for ovine farms worldwide through a decrease in the milk production, milk yield, and cheese-making properties [4,5,6]. Although clinical symptoms are not observed, inflammation and histological lesions are present [7] and animal welfare concerns are considerable [8,9]. Moreover, due to the implication of pathogens with zoonotic potential and the production of enterotoxins [3,10], public health and food safety concerns are not negligible.

The etiological agents of SCM are usually bacteria, some of which exhibit significant pathogenicity and virulence, whereas others are commensal microorganisms that opportunistically cause infections [1,3,7]. The most common group of implicated bacteria is non-aureus staphylococci (NAS), which are mostly coagulase-negative staphylococci (CNS) species, while other species, such as *S. aureus*, streptococci, Enterobacteriaceae, *Pasteurella* spp., *Corynebacterium* spp., and *Pseudomonas aeruginosa*, are regularly found [3,11,12].

Subclinical mastitis demonstrates a total absence of any detectable clinical changes; thus, specific tests in milk are necessary for its identification [1]. Therefore, this condition is frequently underdiagnosed. Currently, the most common tool for screening for ovine SCM is the determination of somatic cells in milk using the California Mastitis Test (CMT). Moreover, microbial culture is the gold standard for identifying etiological agents. However, this approach has certain limitations in terms of the cost and time taken; thus, evaluating and establishing novel auxiliary diagnostic tools, such as biomarkers or other non-invasive tests, are considered beneficial [1,2].

Infrared thermography is a technology that provides real-time, fast, and non-invasive measurements of body temperature in humans and animals, as it allows infrared radiation emitted by the heat source to be converted into the pixel intensity [13,14]. Numerous applications have been described in the literature and adopted in various medical fields [15]. In veterinary medicine, it has mostly been investigated for the detection of pyrexia or localized inflammation encountered in specific pathological conditions [13]. The application of thermal imaging to detect bovine mastitis is a field with significant potential, and several relevant studies have been conducted. In cases of mastitis, prostaglandins are produced through inflammatory processes occurring in the mammary gland and, as a result, the udder’s inner and surface temperatures are increased [14,16]. This allows for the utilization of infrared thermography to detect these variations in the udder temperature, even in subclinical cases or in the early stages of clinical cases, where the symptoms are not detectable [16,17,18]. It has also been evaluated in ovine mastitis with promising results [19,20], but the current data are limited.

Moreover, recent advancements in artificial intelligence have drawn the attention of researchers, including veterinary scientists [21], toward the use of deep learning models. A field with strong potential is the employment of object detection techniques, such as the state-of-the-art YOLO (You Only Look Once) algorithm [22]. YOLO has shown significant promise in veterinary science across various tasks, including both routine farm management and dealing with pathological conditions [23,24,25,26,27]. In this study, we used YOLOv8 to identify the region of interest, which can be further analyzed in a second phase using statistical methods to detect abnormalities, such as higher temperatures.

The objectives of this study were (a) to investigate the incidence, etiologic agents, and characteristics of subclinical mastitis encountered in Lacaune sheep bred in Greece, (b) to preliminarily evaluate the potential of infrared thermography as a fast and non-invasive auxiliary diagnostic tool, and (c) to train and utilize the YOLO algorithm as a preprocessing tool for identifying objects relevant to further analysis.

## 2. Materials and Methods

### 2.1. Investigated Farms and Animals

**Figure 1:**
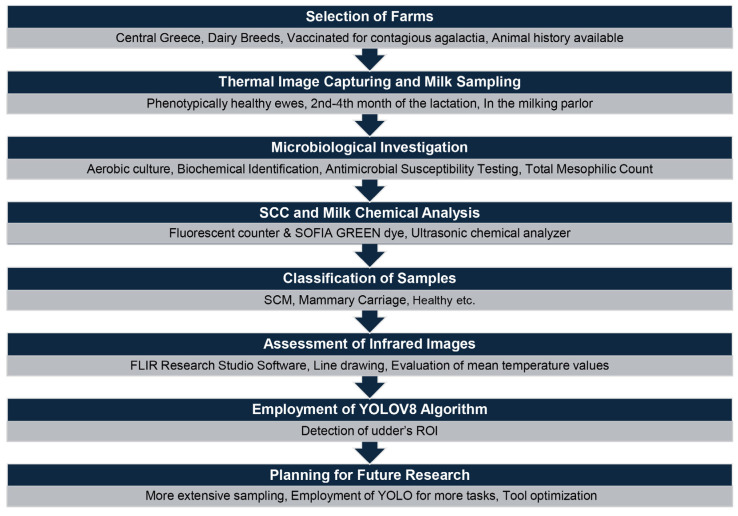
Overview of the procedures carried out in this study.

Crossbred Lacaune and purebred Lacaune sheep from farms located in central Greece were included in this study. Farms were selected according to the following criteria: having an automatic milking system, the availability of a detailed history of previous pathological conditions, treatment for lactating ewes, and vaccination for contagious agalactia. A total of 10 farms were included in this study. All selected ewes were phenotypically healthy and free of any signs of clinical mastitis or other systemic disease. Moreover, all sampled animals were between the 2nd and the 4th month of their lactation period. A total of 330 ewes were sampled. Milk samples were collected between November 2023 and May 2024. A brief overview of the procedures used in this study is shown in Figure 1.

### 2.2. Collection of Milk Samples

Milk samples were obtained once from each farm during routine morning milking according to the guidelines provided by the National Mastitis Council [28]. Two sterile vials were used for each udder half, with and without preservatives, for microbiological and chemical analyses, respectively. Both teats were thoroughly scrubbed with 70% ethyl alcohol using sterile gauze pledgets. The first three streams were discarded, and the necessary quantity (until the respective mark of the puddle disk was reached) was used to perform the CMT. Subsequently, 10–15 mL of milk was aseptically collected in sterile conical vials for microbiological examination. Approximately 40 mL of milk from each mammary gland was added to 50 mL sterile conical vials containing preservative tablets (0.1 g sodium azide, Merck KGaA, Darmstadt, Germany) for chemical analysis and the SCC. Each vial was labeled with the date, animal code, and side of the udder half.

The procedures mentioned above were carried out every time by the same experienced personnel (S.T. and P.K.) to obtain objective CMT results and similar sampling conditions. The milk samples were immediately stored at 1–5 °C and transferred to the laboratory within 2 h for further investigation.

### 2.3. Microbiological Examination

#### 2.3.1. Aerobic Culture and Microorganism Identification

Each milk sample was left for 3–5 min at room temperature before handling. Then, it was gently shaken for 15–20 s and 1 mL of it was pipetted into a sterile tube containing 9 mL of maximum recovery diluent (MRD, Oxoid, Basingstoke, UK). The suspension was thoroughly vortexed (at high speed for 10 s), and a second tube was used to prepare the next decimal dilution. Subsequently, 100 μL of each decimal dilution (representing the 10^−2^ and 10^−3^ dilutions) and 10 μL from the second tube (representing the 10^−4^ dilution) were distributed on the surface of Petri dishes of sheep blood agar, MacConkey agar, and mannitol salt agar (Oxoid, Basingstoke, UK) in duplicate, using sterile spreaders. The plates were then incubated at 37 °C for 24 h. In cases of limited or no growth during the initial evaluation, the incubation period was extended to 48 h or 72 h to obtain well-grown colonies or to check for the presence of bacteria with prolonged incubation periods (such as *Nocardia* spp.).

The identification of the detected pathogens was accomplished through morphological characteristics, Gram staining, and biochemical characterization using commercial kits and associated software (API^®^ Staph, API^®^ Strep, API^®^ 20E, API^®^ 20NE, apiweb™ v1.4.1-3; Biomérieux, Lyon, France). In the cases of non-bacterial pathogens (*Candida* spp.) being detected, identification was performed using matrix-assisted laser desorption/ionization time-of-flight mass spectrometry (MALDI-TOF MS).

The population of identified bacterial pathogens was calculated using a horizontal enumeration technique [29], including all the sheep blood agar plates of the respective sample with 10–300 colony-forming units (cfu). If growth of more than 300 cfu occurred in both plates of the 10^−4^ dilution, the population was defined as >3 × 10^6^ cfu/mL. For *Proteus mirabilis*, MacConkey agar plates were used instead of sheep blood because of swarming.

The criteria applied for intramammary infection (IMI) discrimination were described in previous studies [1,30]. In particular, cases of pure culture growth with a mean value of ≥10 colonies in the 10^−2^ dilution (corresponding to ≥1000 cfu/mL in milk) were identified as IMI. All cases of IMI with a respective SCC value of >500,000 per mL, based on the Lactoscan SCC results (Section 2.4), were defined as SCM, while those with an SCC < 250,000 per mL were classified as having mammary carriage (MC), as previously described [11,31].

Regarding the classification of the remaining cases, some additional criteria were established for this study, as variable SCC and bacteriological thresholds have been suggested in the literature [2,31,32,33,34]. The criteria used are listed in Table 1. In particular, all samples with SCC values between 250,000 and 500,000 were further investigated, as a threshold of 250,000 was suggested by some authors [2,32,33]. Moreover, considering the confidence intervals for the colony count technique [35] and the fact that the secretion of bacteria in milk could be altered over time [36], the growth of a species with a mean value of 7–9 cfu in the 10^−2^ dilution (based on the colony morphology) was also considered in the case of high SCC values. Some authors have suggested lower colony count thresholds for discriminating an infected mammary gland [37]. The criteria included in Table 1 were evaluated for the sufficient interpretation of all samples after the thorough examination of the available results.

#### 2.3.2. Total Mesophilic Count (TMC)

The standard pour plate technique for colony counting was performed at 30 °C [29]. The Petri dishes were incubated for 72 h, and calculations were performed using well-established criteria [35].

#### 2.3.3. Antimicrobial Susceptibility Testing

The disk diffusion method was used for the following antibiotics: ampicillin, amoxicillin–clavulanic acid, cefoxitin, ceftiofur, cephalexin, ciprofloxacin, enrofloxacin, erythromycin, gentamicin, marbofloxacin, novobiocin, sulfamethoxazole–trimethoprim, and tetracycline. All procedures, disk contents, and evaluated clinical breakpoints were in accordance with the relevant veterinary and human Clinical & Laboratory Standards Institute (CLSI) documents [42,43]. As there are no ovine-specific breakpoints, the approach for their selection was based on the tested microorganism and relevant CLSI guidelines [44], and the order was as follows: intramammary infection-specific, cattle-specific, other animal-specific, and human-specific breakpoints.

### 2.4. Somatic Cell Count

The SCC was carried out using a direct fluorescent image low-magnification microscopic recognition method, using a LACTOSCAN SCC counter (Milkotronic Ltd., Nova Zagora, Bulgaria), four-chamber disposable chips, and microtubes containing SOFIA GREEN lyophilized dye (Milkotronic Ltd., Nova Zagora, Bulgaria). Before the test, milk samples were heated to 40 °C, cooled to 20 °C, vortexed to uniformly distribute fat, and subsequently diluted with water at a ratio of 1:1 before the test (as indicated in the respective manual, since a fat content of >5% is expected for ovine milk). All procedures were performed according to the manufacturer’s instructions.

### 2.5. Milk Chemical Analysis

The chemical analysis of milk was performed using an ultrasonic milk analyzer (Lactoscan FARM ECO, Milkotronic Ltd., Nova Zagora, Bulgaria) according to the manufacturer’s instructions. Before the test, all the samples were slowly heated to 35–40 °C in a water bath to dissolve and distribute the fat content uniformly.

### 2.6. Thermal Imaging and Analysis

Three to five thermal images were received from each animal before performing the CMT. All images were taken at a distance of 70 cm from the posterior surface of the udder using a FLIR E96 24° camera (Teledyne FLIR LLC., Wilsonville, OR, USA). The procedures were carried out in a milking parlor to avoid the effects of climatic conditions (wind, rain, etc.). Environmental conditions (temperature and humidity) during sampling were recorded using a COMET U3120 Datalogger (COMET SYSTEM s.r.o., Roznov pod Radhostem, Czech Republic). Images (jpg files) were processed using FLIR Research Studio (version 3.0.1) software (Teledyne FLIR LLC., Wilsonville, OR, USA). The analysis included only high-quality images in which both udder halves were clearly visible and factors that could affect the udder skin temperature, such as wounds, scars, and dirt, were absent. From each of the selected animals, only one image was included, according to the previous criteria and file size, to assess those with the best quality.

The line tool was mostly used in the analysis. Numerous approaches were tested during the initial assessment (e.g., the comparison of maximum udder temperatures or mean temperatures in specific udder areas using the elliptical tool), with no significant efficiency in separating healthy from infected udder halves in the investigated group. Moreover, through a thorough examination of SCM images, it was observed that the temperature of the upper region of the udder was affected mostly by the proximity to the body of the ewe, the way the animal stood during the shot, and perhaps other factors irrelevant to mastitis (length of the wool, etc.). Therefore, we focused on the region between the teats, which is more uniformly exposed in all animals and easier to define. The technique described below is relatively simple and demonstrates discriminative potential; therefore, it was included in this study.

Initially, to reduce the influence of external (climatic conditions, the time of milking, the layout and ventilation of milking parlor, etc.) and animal-specific factors (the udder anatomy, age, nutritional status, previous pathological conditions, etc.) that were not directly related to the investigated udder inflammation process, animals with unilateral SCM and an expected considerable diversity in the health status between the right and left mammary glands were selected. For this reason, all udder halves were classified in five categories according to their SCC value (1: <250,000; 2: 250,000–500,000; 3: 500,000–1,500,000; 4: 1,500,000–5,000,000; 5: >5,000,000), according to the thresholds evaluated during the microbiological investigation (250,000 and 500,000), and according to the empirical SCC ranges corresponding to positive CMT results (CMT: 1 < 1,500,000; CMT: 2 < 5,000,000; CMT: 3 > 5,000,000), which were used to classify SCM cases according to the severity of the inflammation process. Subsequently, only animals with a difference of ≥2 SCC classes between the two halves were assessed. Fifty-seven animals were included in this study (the unilateral SCM group).

**Figure 2:**
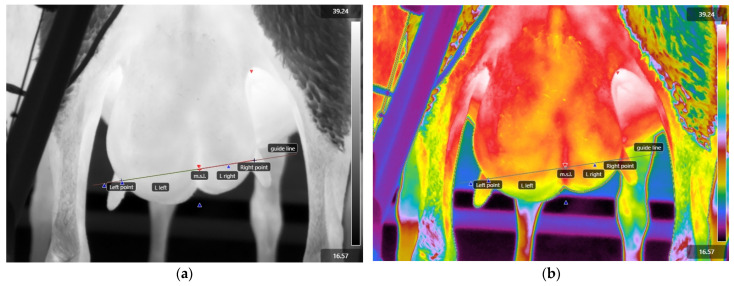
Evaluation of thermal images: (**a**) manual drawing of L lines for each mammary gland (grayscale); (**b**) lines on a thermal image of a gland using a rainbow high-contrast filter.

Using the line tool of the FLIR Research Studio (version 3.0.1) software (Teledyne FLIR LLC., Wilsonville, OR, USA), a line was drawn between the teats, uniting the observed middles of their bases, which were defined as the left and right points according to the udder’s side (Figure 2). This line was then divided into two parts (one for each half) in the median suspensory ligament (m.s.l., Figure 2). These lines were defined as the L_L_ and L_R_ for the left and right udder halves, respectively. The mean temperature values (T_L_ and T_R_) of the measurements corresponding to each line (L_L_ and L_R_, respectively) were obtained by using the aforementioned software.

These procedures were also performed for a second group of 60 healthy animals, which were selected based on the absence of bacterial growth and an SCC value of <250.000 per mL in the milk samples of both udder halves (healthy group).

In addition, more SCM cases were included in the third group (the bilateral SCM group). Animals with bilateral SCM and similar SCC values in both udder halves (a difference of <2 classes according to the categorization mentioned above) were assessed. A total of 21 cases were identified. All relevant temperature data are included in Supplementary Table S1.

### 2.7. Usage of YOLOv8 Algorithm for Identification of Teat Location

The selected methodology consisted of three distinct phases: preprocessing, fine-tuning, and testing. Each phase played a crucial role in ensuring the effectiveness of this object detection model for identifying specific features in thermal images.

#### 2.7.1. Preprocessing

In the preprocessing phase, the dataset of thermal images was carefully curated to optimize it for training and testing the object detection model. A total of 174 thermal images were initially selected, in which at least one teat was clearly visible. Additionally, images in which one or both teats were partially obscured but still identifiable were included. This approach was essential because it allowed us to accurately draw a line between the two teats.

Following this selection process, 136 images were used for training and 23 for testing. The selected images were annotated using LabelImg (https://github.com/HumanSignal/labelImg, accessed on 12 August 2024) to ensure that the model could effectively learn from the dataset. LabelImg is a versatile annotation tool that enables users to define specific regions within images by drawing bounding boxes around them. For this task, bounding boxes were drawn around the teats, with the center of each box positioned at the base of the teat. The class was labeled as “teat”. The coordinates of these annotated regions were extracted for further analysis, providing the data necessary to train our model.

#### 2.7.2. Fine-Tuning Process

The fine-tuning process involved utilizing the images and their corresponding coordinates within the YOLOv8 algorithm. A total of 136 training images were loaded into the YOLOv8 framework, which allowed the model to distinguish teats from the other components of the thermal images.

During this phase, fine-tuning for 100 epochs was conducted, and the model parameters were systematically adjusted to enhance accuracy. Early stopping with a patience of 10 epochs, monitoring the mean Average Precision (mAP), was employed. A grid search approach was followed to define the optimal hyperparameters for our model, resulting in the selection of the Adam optimizer, a learning rate of 0.11, a weight decay of 0.0045, and a batch size of 16. The extensive training aimed at improving the model’s ability to detect and classify targeted features effectively. The model was trained with a range of augmentations to enhance robustness and diversity. Brightness variations (±40%) introduced lighting diversity, while spatial variation was achieved through geometric transformations, including translation (±10%), scaling (±50%), and horizontal flipping (50% probability). Mosaic augmentation was applied to enrich the dataset, and random erasing (40% probability) added resilience to occlusions. Training completed in 340.8 s on an NVIDIA GRID RTX6000P-6Q GPU, with early stopping triggered at epoch 80.

#### 2.7.3. Testing

In the testing phase, we evaluated the performance of the YOLOv8 algorithm by using two primary methods. First, a 5-fold cross-validation was implemented on the training set to fine-tune the parameters of the YOLOv8 model. This technique involved dividing the dataset into five subsets and using each subset in turn for validation, whereas the remaining four subsets were used for training.

After completing the cross-validation, the performance of YOLOv8 fine-tuned on the training set using 23 test images was evaluated. This final step allowed us to determine the accuracy and reliability of the model in detecting targeted features in previously unseen images. The results from both the cross-validation and the testing phase were analyzed to identify areas for potential improvement in the model performance.

#### 2.7.4. Drawing the Line

Given that our model was designed to predict the locations of sheep teats, we implemented a simple approach to accurately draw a line between them. Specifically, we first identified the center points of the bounding boxes that the YOLOv8 model successfully detects. By calculating the coordinates of these centers, a line could be drawn connecting one center to the other.

### 2.8. Statistical Analysis

The obtained temperature data from all three categories (healthy animals, unilateral SCM, and bilateral SCM) of the investigated images were tested for normality using the Kolmogorov–Smirnov normality test (K-S). This assumption was satisfied in all the cases; thus, no further transformation was essential.

A dependent *t*-test for paired samples was performed to compare the mean temperature values of the left (T_L_) and right (T_R_) udder halves in each group (significance level set at *p* < 0.05, two-tailed hypothesis).

An unpaired *t*-test was performed to compare temperature values between the groups of bilateral SCM and healthy animals (significance level set at *p* < 0.05, two-tailed hypothesis).

ROC analysis was performed to investigate whether a cut-off point could be established that would allow for satisfactory sensitivity and specificity for the discrimination of infected from uninfected animals. Analysis was performed on the data of the healthy group vs. bilateral SCM as well as the data on the infected vs. uninfected udder halves in the unilateral SCM group.

A comparison of the TMC values between the five SCC groups and the three bacteriological status groups was performed using a one-way ANOVA followed by Tukey’s HSD. The same statistical tools were used to compare chemical parameters (fat, lactose, proteins, total solids) between the five SCC groups and the three SCM Staphylococci groups.

All analyses were performed using IBM SPSS Statistics for Windows, Version 29.0.2.0 (IBM Corp., Armonk, NY, USA).

## 3. Results

### 3.1. Incidence of Subclinical Mastitis and Isolated Pathogens

In total, 660 milk samples were obtained from 330 ewes. During the microbiological investigation, bacterial (or yeast) growth was observed in 181 (27.4%) milk samples. A total of 184 bacterial and yeast strains were isolated from these samples, since three cases of mixed infections were identified. These samples were obtained from 127/330 ewes (38.5%). Moreover, 157 udder halves were identified as having SCM, corresponding to 122/330 ewes (37.0%). Of these cases, 35 (28.7%) were bilateral and 87 (71.3%) were unilateral. A group of 24 samples from 23 ewes was classified as indicating mammary carriages.

Data on the incidence of implicated bacteria and yeasts in SCM and MC cases are presented in Table 2.

As shown in Table 2, Gram-positive cocci were prevalent among the identified pathogens, representing 93.8% (150/160) of the microorganisms isolated from SCM cases. The majority were staphylococci and were mostly coagulase-negative species. Detailed data regarding the staphylococcal SCM cases are presented in Table 3.

The prevalent species was *S. epidermidis*, which was isolated from 40 milk samples from 32 ewes. Other regularly encountered species included *S. chromogenes* (21), *S. simulans* (17), *S. hyicus* (17), and *S. caprae* (13). The mean SCC values of the respective milk samples were similar for *NRCNS* and *CPS* and slightly higher for *NSCNS.* Regarding the implicated species, *S. simulans* and *S. haemolyticus* were associated with higher somatic cell and bacterial cell counts in the respective milk samples. Moreover, a higher SCC was observed in the three *S. aureus* cases, while the population of this pathogen was relatively low.

### 3.2. Antimicrobial Resistance

Considerable antibiotic resistance was identified for ampicillin, sulfamethoxazole–trimethoprim, and tetracycline with rates of 28.6% (52/182), 23.6% (40/169), and 29.7% (54/182), respectively. Novobiocin resistance was detected in 11.9% (19/159) of the tested bacteria, corresponding mostly to novobiocin-resistant CNS species (*S. xylosus*, *S. sciuri*), *Micrococcus* spp., and Corynebacteria. The rates for all other antibiotics were below 5% (excluding intrinsic resistance mechanisms, e.g., aminoglycoside resistance in enterococci).

### 3.3. Total Mesophilic Count

Data on the total mesophilic counts per group of milk samples are presented in Table 4.

The total mesophilic counts generally increased in milk samples with a higher SCC (one-way ANOVA for the five SCC groups; F = 33.260624; *p* < 0.001). Post hoc comparisons using the Tukey HSD test showed that the mean TMC values for the SCC groups above 500,000 per mL were significantly higher than those for the SCC groups below 500,000 per mL. The differences between the 500,000–1,500,000, 1,500,000–5,000,000, and >5,000,000 groups were not statistically significant. Furthermore, with respect to the bacteriological status of the milk, samples with both mammary carriage and bacterial subclinical mastitis demonstrated significantly higher counts than samples with no growth in an aerobic culture (one-way ANOVA for the three bacteriological status groups, followed by Tukey’s HSD; F = 90.636621; *p* < 0.001).

### 3.4. Chemical Analysis

Five milk samples were excluded from the analysis due to a limited clot presence that was not identified macroscopically during sampling. Four of them were identified as IMI cases (*Pasteurella* sp., *Proteus mirabilis*, *S. aureus*, *S. simulans*) and all of them demonstrated increased SCC values (>4,000,000 per mL). Data regarding the chemical composition of the remaining samples per category of SCC and the isolated pathogens are presented in Table 5.

An increase in the SCC was generally correlated with lower concentrations of fat, lactose, protein, and total solids. More specifically, a decrease in fat was statistically significant for SCC > 5,000,000 per mL in comparison to the other SCC groups (one-way ANOVA for the five SCC groups followed by the Tukey HSD; F = 5.000077; *p* < 0.001). For lactose and protein, SCC groups below 500,000 per mL demonstrated a significantly higher percentage in comparison to SCC groups above 1,500,000 per mL (one-way ANOVA for the five SCC groups followed by the Tukey HSD, F = 7.121005 and *p* < 0.001 and F = 7.581481 and *p* < 0.001, respectively). A statistically significant decrease in the total solids was detected in the SCC > 5,000,000 group compared with the other four groups, which did not show any statistically significant difference between them (one-way ANOVA for the five SCC groups followed by Tukey’s HSD; F = 8.119827; *p* < 0.001). Regarding SCM staphylococcal infections, no statistically significant differences were detected for the fat, lactose, protein, and total solids composition between the CPS, NSCNS, and NRCNS groups (one-way ANOVA; *p* = 0.626, *p* = 0.492, *p* = 0.506, and *p* = 0.815, respectively).

### 3.5. The Evaluation of Infrared Thermography as a Diagnostic Tool

According to the K-S test, the obtained temperature (T) values were normally distributed in all three investigated groups of unilateral SCM, bilateral SCM, and healthy animals (K-S values of 0.12715, 0.18756, and 0.11174 with *p* = 0.24708, 0.40126, and 0.41203, respectively).

The paired samples *t*-test revealed a statistically significant difference in the group of unilateral SCM cases *(t* = 7.5849; *p* < 0.001), with the T values being significantly higher (a mean increase of 0.59 °C and SD of 0.58 °C) in the SCM udder halves. The mean T values were 34.69 °C (SD = 1.60 °C) and 34.10 °C (SD = 1.77 °C) for the SCM udder halves and the healthy udder halves, respectively. In contrast, no statistically significant difference in the T values was found between each animal’s udder halves in the bilateral SCM group *(t* = 1.557; *p* = 0.1353) and in the healthy group *(t* = 0.047; *p* = 0.9628).

The mean value of all T (T_L_ and T_R_) measurements was 35.45 °C with an SD of 1.64 °C in the bilateral SCM udders and 34.69 °C with an SD of 1.27 °C in the healthy udders. By comparing these two groups (unpaired *t*-test), this difference was found to be statistically significant *(t* = 3.078; *p* = 0.0024), demonstrating a significantly higher T value in the bilateral SCM udders than in the healthy ones.

Even though a statistically significant difference was detected when comparing the healthy vs. the bilateral SCM group and the infected vs. the uninfected udder halves in the unilateral SCM group, ROC analysis did not reveal a cut point with an acceptable diagnostic sensitivity and specificity for detecting SCM (AUC = 0.674 with CI95% = 0.573–0.775 and AUC = 0.618 with CI95% 0.514–0.722).

### 3.6. Teat Detection and Line Drawing Utilizing YOLOv8 Algorithm

After performing hyperparameter tuning through a grid search to determine the optimal parameters, we evaluated the performance of the YOLOv8 model on a test set comprising 23 images, which yielded promising results. Specifically, the model achieved a precision of 91.27%, a recall of 95.65%, an mAP@50 of 96.94%, and an mAP@50–95 of 54.74%. These metrics demonstrate that the model effectively addresses the challenge of detecting sheep teats, despite certain limitations within the dataset.

One key limitation involved the images in the test set where only one teat was visible. In such cases, it was impossible to establish a line connecting both, which was a crucial component of our approach. To accurately assess the ability of our approach to draw this line, we excluded images with only one visible teat, reducing the test set to 19 images.

Upon re-evaluating the performance on this subset of images, an accuracy of 84% was achieved for drawing a line between the teats. This result suggests that while the model performs well in teat detection, further refinement is needed to improve its ability to consistently detect both teats and estimate their positions for line-drawing tasks, particularly in more complex or occluded scenarios.

**Figure 3:**
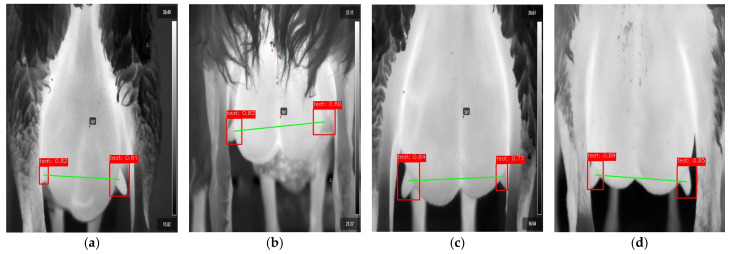
Teat detection in thermal images, including line drawings. Analysis of four thermal image instances (confidence levels produced by the model for detecting the teats are displayed).

Finally, in Figure 3, four instances of thermal images are presented where one or both teats are partially obscured or only partially visible. Despite these visual obstructions, the YOLOv8 model successfully detected their locations. After detection, we established and drew a line connecting the detected teats in each image.

## 4. Discussion

### 4.1. Prevalence of Subclinical Mastitis and Implicated Bacteria

The results of this study demonstrate a high prevalence of subclinical mastitis in dairy sheep farms in Greece, the predominance of coagulase-negative staphylococci in relevant cases, and considerable rates of resistance against specific, widely used antimicrobial agents. Moreover, the potential of infrared thermography as an auxiliary diagnostic tool is highlighted, although more samples and the employment of AI tools in more demanding tasks are essential to optimize the respective approach and improve its efficiency. The rate of subclinical mastitis in the investigated sample was 37.0%, with 26.4% being unilateral cases and 10.6% being bilateral cases. Similar rates have been reported in numerous previous studies [3,30,36,45,46], whereas lower rates have been identified by other authors [37,47]. The regular detection of infected animals at noteworthy rates indicates the importance of SCM in lactating ewes and the necessity for proper monitoring and control at the farm level. It has also been suggested to be the primary cause of milk drop syndrome in dairy sheep in Greece [9,45].

Regarding the implicated bacteria, CNS were predominant (115/160, 71.9%). This was anticipated for SCM cases, since over 80% of the relevant studies worldwide report their prevalence [12] at rates of up to 70% of bacterial isolates [11]. The most commonly encountered species was *S. epidermidis*, while *S. chromogenes*, *S. simulans*, *S. caprae*, *S. capitis*, and *S. xylosus* were regularly detected. This is in accordance with previous studies, since these species are frequently isolated from subclinical diseases and occasionally from clinical cases [3,9,12,30]. Coagulase-positive staphylococci were obtained from 21 cases, of which 17 corresponded to *S. hyicus.* Although this species is usually detected at lower rates [12,30], some authors have reported a higher incidence [41,48,49,50]. Major pathogens (*S. aureus*, *S. agalactiae*, *Pasteurella* sp.) were identified in a limited number of cases, which was anticipated since they are mostly associated with clinical mastitis [9,51].

In reference to antibiotic resistance, significant rates of acquired resistance were documented for ampicillin (28.6%), sulfamethoxazole–trimethoprim (23.6%), and tetracycline (27.9%), whereas all other antibiotics were highly effective in vitro. The rates for ampicillin and tetracycline were similar to those usually reported in the relevant literature; however, they were relatively higher than the mean values of studies originating from Europe [11,12]. The rate of sulfamethoxazole–trimethoprim (SMX/TMP) was noteworthy because in most studies, much lower percentages of resistant bacteria were identified [12]. Nonetheless, this rate was affected by the detection of ten cases of SMX/TMP-resistant *S. simulans* in six ewes in a single farm and six cases of SMX/TMP-resistant *S. hyicus* in four ewes on another farm. Therefore, the actual distribution of resistance among the bacterial pathogens was rather low. The fact that antibiotic susceptibility testing demonstrated high percentages of resistance exclusively to these agents could be associated with the wide usage of the respective classes of antibiotics in food-producing animals in the country [52] and the consequential selection pressure.

### 4.2. Association Between Subclinical Mastitis and Milk Characteristics

No significant variations in the SCC of staphylococcal SCM cases caused by different species were identified in this study, except for *S. simulans*, *S. haemolyticus*, and *S. aureus*, where higher values were detected. Similar results were reported in other studies [36,53,54]. However, lower values in cases of CNS infection have been identified in some studies [55,56,57]. This difference could be a result of different classification criteria for the selection of the included cases, different breeds, or different lactation stages during sampling. *S. simulans* is a CNS species regularly implicated in SCM [11,12], while it has also been associated with cases of clinical mastitis [9]. Specific virulence factors or the ability to produce biofilms [19,58,59] could constitute elements of higher pathogenicity in some strains. Finally, novobiocin-susceptible CNS species demonstrated a slightly higher mean SCC than novobiocin-resistant ones. This is in accordance with previous reports [40], but the variation in this study is mostly a result of the comparatively greater values observed in *S. simulans* cases, ten of which originated from a single farm.

In reference to the chemical analysis of the milk, a decrease in the lactose, fat, and protein content was identified in cases of an increased SCC, which seemed to be greater in values > 5,000,000 per mL. The decrease in lactose has been documented by almost all authors in the relevant literature [4]. In addition, it is considered a potential indicator of SCM [60]. However, the results for the fat content and proteins are controversial among different studies, and most authors have reported an increase in whey proteins [4]. Moreover, no statistically significant variations in the composition of the milk samples were documented between the different staphylococcal species. However, the low number of strains isolated from the NRCNS and CPS groups does not allow for solid conclusions. The type of implicated pathogen has been reported to be a crucial factor associated with the extent of the milk yield loss, according to previous studies [4,6,40].

### 4.3. Assessment of Infrared Technology and YOLOv8 Algorithm

During the analysis of the infrared images, significantly higher mean temperatures were observed in the examined regions of the udder halves with SCM than in the uninfected mammary glands. Accordingly, in a previous study, udders with subclinical mastitis exhibited higher temperatures than both healthy and clinical mastitis cases [19]. However, the detected variations were generally low (<0.5 °C), similarly to our results. Thus, as demonstrated by ROC analysis, it is difficult to define a threshold based only on the temperature value to detect SCM cases. Many factors that may affect the udder surface temperature must be considered, and further investigation is needed. Statistically significant findings detected when comparing SCM and healthy groups demonstrate future potential.

In a recent study in Greece, an advanced algorithmic approach was proposed, in which the mean temperature values and standard deviations were also evaluated [20]. The classification accuracy was high (84%), highlighting the potential for the implementation of artificial intelligence (AI) tools in the investigation.

Deep learning techniques, particularly YOLO, have been applied to various sheep-related tasks. Convolutional neural networks (CNNs) have been utilized for sheep identity recognition, achieving high accuracy while remaining suitable for edge devices [61]. Similarly, YOLOv3 has been employed for fast and accurate sheep face recognition [62]. YOLO has also been used to detect sheep’s behavior and posture, as proposed by Deng et al. [63]. The Light Attention YOLO model, which integrates an attention depth-separable module with CSPDarknet and PAFPN, enhances the real-time detection and counting of sheep by incorporating the CBAM attention module into the fourth and fifth layers of the backbone network [64].

To the best of our knowledge, none of the aforementioned approaches have addressed the problem of recognizing sheep teats in a context similar to that in this study. Despite working with a small and noisy dataset where teats were occasionally only partially visible, our results demonstrate the effectiveness of this approach, yielding promising outcomes. Building on these promising results, our work demonstrates the potential of deep learning models to overcome the challenges posed by imperfect datasets such as obscured teats in thermal images. The success of YOLO in accurately identifying teat locations even under these conditions suggests the robustness of this model for practical applications in the field of veterinary science. Moreover, this research lays the groundwork for further investigation into the use of deep learning for thermal image analysis in livestock, which could extend beyond object detection to other diagnostic and health monitoring tasks [65].

### 4.4. Study Limitations and Future Perspectives

This study has certain limitations. Initially, the microbiological examination included only certain pathogens, whereas ovine mastitis could be mediated by lentiviruses or other non-bacterial agents [3,9]. Moreover, the investigation did not include the molecular characteristics of the implicated pathogens, such as the detection and expression of virulence genes, which could provide crucial information regarding their pathogenicity. Furthermore, the approach in the evaluation of thermal images (at this preliminary stage) was simple and was accomplished by manual tests using data obtained from the relevant software. The images could not be analyzed as a whole at this stage of the research, and a limited amount of available infrared data was included in the analysis. However, a large number of milk samples and high-quality thermal images were assessed, and the approach was independent of several confounding variables.

Regarding the application of the YOLOv8 algorithm, one significant limitation of this study is the small and noisy dataset currently available for analysis. The dataset comprised thermal images where the visibility of the teats was inconsistent—sometimes only partially visible—sometimes one teat was hidden, or in some cases, both teats were entirely obscured or absent. This inconsistency posed a significant challenge for the methodology, particularly because the limited number of clear instances prevented us from fine-tuning the YOLOv8 algorithm with the necessary precision. Without a diverse and comprehensive dataset, the model struggled to generalize well, which impacted its ability to accurately detect and analyze key features, such as the teat location, across varying conditions. Moreover, the noisiness of the dataset introduced an additional layer of complexity because thermal images can be affected by a variety of factors, such as temperature fluctuations and visual obstructions.

In reference to future perspectives, extended sampling with the simultaneous evaluation of the proposed measurements in numerous high-quality images and in the field could provide useful information regarding the approach’s feasibility in routine veterinary practice. Additionally, the results of the analysis of the thermal images should be associated with more factors, such as the implicated pathogen species, their virulence, the severity of the inflammation process in the examined udder, the animal’s characteristics (age, the stage of the lactation period, previous history), milk characteristics, etc., to determine the association of each one with temperature variation patterns and define suitable criteria for detecting as many SCM cases as possible. More data should be obtained from thermal images and included in the analysis. Finally, the implementation of AI tools could be significantly improved by increasing the number of images and ensuring that they capture a broader range of scenarios, including variations in visibility, lighting, and teat positioning. Advanced techniques such as data augmentation can be employed to artificially expand a dataset by introducing variations in existing images, thus providing an algorithm with more diverse training data [66].

## 5. Conclusions

Subclinical mastitis is common in phenotypically healthy dairy ewes in Greece. The quality of the provided milk is significantly affected, and concerns regarding animal health and welfare have emerged. Staphylococci and coagulase-negative species were the predominant implicated pathogens. The resistance rates to specific, widely administered antibiotics are noteworthy. Infrared thermography is a promising tool for the rapid and early detection of ovine SCM. The analysis of thermal images of the udder, with a concurrent evaluation of temperature patterns, could be utilized to identify a significant percentage of infected animals. More extensive sampling and evaluations of AI applications are essential to optimize the technique and establish an appropriate auxiliary diagnostic tool.

## Figures and Tables

**Table 1:** Classification criteria applied in this study for the microbiological evaluation of milk samples.

Classification	SCC	Different Colonies	Population (Cfu in the 10^−2^ Dilution)	Extra Criteria ^1^
Healthy	<250,000	No growth		
Healthy		One or more	Total < 7	
Healthy (MC)		One	≥7	
Contamination		Two or more	Total ≥ 7	
Healthy	250,000–500,000	No growth		
Healthy		One or more	Total < 7	
Healthy (MC)		One	≥7	(PMNs + LYMs) < 75%
SCM				(PMNs + LYMs) ≥ 75%
Healthy (MC)		Two	One with ≥7 ^2^	(PMNs + LYMs) < 75%
SCM				(PMNs + LYMs) ≥ 75%
Contamination			≥7 for both	(PMNs + LYMs) < 75%
SCM (mixed infection) ^3^				(PMNs + LYMs) ≥ 75%
Contamination			<7 for both and total > 7	
Contamination		Three or more		
SCM Other	≥500,000	No growth		
SCM Other		One or more	Total < 7	
SCM		One	≥7	
SCM		Two	One with ≥7 ^2^	
SCM (mixed infection) ^3^			≥7 for both	
Contamination			<7 for both and total > 7	
Contamination		Three or more		

SCM: subclinical mastitis; MC: mammary carriage; PMNs: polymorphonuclear leukocytes; LYMs: lymphocytes; SCM Other: subclinical mastitis of other etiology (e.g., viral, *Mycoplasma*, traumatic, etc.). ^1^ In the case of bacterial growth and an SCC value between 250,000 and 500,000 per mL, samples were further investigated, as some authors suggest a threshold of 250,000 per mL or even lower counts [38]. Microscopic examination and differential somatic cell counts were performed as previously described [3,30]. The percentage of polymorphonuclear leukocytes (PMNs) and lymphocytes (LYMs) in the total leukocytes was calculated in at least 10 fields, and the threshold was defined as 75% [39] for the discrimination of SCM (≥75%) from MC (<75%). ^2^ The prevalent species was defined as the etiological agent. ^3^ Mixed infections of the mammary glands have been previously described [40,41].

**Table 2:** Microorganisms isolated from SCM and MC cases.

Isolated Species	Subclinical Mastitis	Mammary Carriage
*Aerococcus viridans*	1	1
*Bacillus licheniformis*	0	1
*Candida* spp. ^1^	2	0
*CNS* (total)	115	13
*CPS* (total)	21	0
*Corynebacterium* spp.	1	3
*E. coli*	4	0
*Enterobacter cloacae*	1	0
*Enterococcus* spp. ^2^	6	3
*Gemella haemolysans*	0	1
*Micrococcus* spp.	4	1
*Pasteurella* sp.	1	0
*P. mirabilis*	1	0
*Streptococcus* spp. ^3^	3	1
Total	160 ^4^	24

^1^ *C. parapsilosis* (n = 1), *C. kefyr* (n = 1); ^2^ *E. durans* (n = 3), *E. faecalis* (n = 5), *E. faecium* (n = 1); ^3^ *S. agalactiae* (n = 1), *S. pluranimalium* (n = 1), *S. uberis* (n = 1), *Streptococcus* sp. (n = 1); ^4^ three cases of mixed infection were identified and thus, 160 bacterial and yeast strains were obtained from 157 SCM cases.

**Table 3:** Number of cases per isolated species, mean SCC, and cfu/mL of milk for SCM cases caused by staphylococci.

Isolated Species	SCM Cases ^1^	Infected Ewes ^2^	Mean SCC ^3^ (CI95%)	Mean Cfu/mL ^3^ (CI95%)
*CNS* (total)	115	89	3,343,655 (2,938,677–3,748,633)	15,558 (12,150–18,966)
*NSCNS* ^4^ (total)	106	80	3,370,017 (2,926,635–3,813,399)	16,687 (13,258–20,116)
*S. epidermidis*	40	32	2,054,656 (1,360,611–2,748,701)	18,140 (12,754–23,526)
*S. chromogenes*	21	18	1,850,482 (1,283,314–2,417,650)	6590 (2722–10,458)
*S. simulans*	17	12	7.761.782 (4,994,378–10,529,186)	27,058 (12,307–41,809)
*S. caprae*	13	11	3,945,728 (1,417,682–6,473,774)	9100 (4738–13,462)
*S. capitis*	9	7	2,872,545 (1,093,504–4,651,550)	27,911 (12,457–43,365)
*S. haemolyticus*	3	3	7,667,222	21,800
*S. warneri*	3	2	1,384,781	3400
*NRCNS* ^5^ (total)	9	9	3,039,028 (670,942–5,407,114)	2267 (1026–3508)
*S. xylosus*	8	8	3,245,073 (562,577–5,927,569)	2325 (983–3667)
*S. sciuri*	1	1	1,390,669	1800
*CPS* (total)	21	19	3,086,724 (2,132,996–4,040,452)	22,015 (11,294–32,736)
*S. hyicus*	17	15	2,750,195 (1,673,575–3,826,815)	25,488 (13,116–37,860)
*S. aureus*	3	3	4,993,722	2333
*S. schleiferi*	1	1	1,132,720	1300

^1^ Total identified SCM cases in udder halves, caused by the relevant species; ^2^ animals corresponding to the SCM cases of the previous column, infected unilaterally or bilaterally. In the latter case, both udder halves were accounted for as SCM cases. ^3^ Of the relevant group of SCM cases; ^4^ novobiocin-susceptible CNS species; ^5^ novobiocin-resistant CNS species.

**Table 4:** Total mesophilic count ^1^ values per group of milk samples.

Group of Samples		TMC (CI95%)
SCC Groups	SCC *<* 250,000	7061 (4857–9265)
	SCC: 250,000–500,000	16,681 (11,529–21,833)
	SCC: 500,000–1,500,000	46,527 (33,651–59,403)
	SCC: 1,500,000–5,000,000	55,073 (40,200–69,946)
	SCC > 5,000,000	57,012 (31,437–82,587)
Bacteriological Status Groups ^2^	No bacterial growth (healthy and SCM Other)	6464 (4675–8253)
	Mammary carriage	43,773 (25,290–62,256)
	SCM	54,679 (41,694–67,664)

^1^ Total mesophilic count: the number of viable microorganisms that grow under aerobic conditions and moderate temperatures per mL of the investigated sample. ^2^ According to the classification presented in Table 1.

**Table 5:** Milk composition per group of samples, regarding SCC and bacterial infection.

Group of Samples		Mean Values, % (CI95%)			
		Fat	Lactose	Protein	Total Solids
	SCM ^1^	6.60 (6.38–6.82)	4.68 (4.62–4.74)	4.91 (4,86–4.96)	17.02 (16.76–17.28)
SCC Groups ^2^	SCC < 250,000	6.77 (6.59–6.95)	4.85 (4.81–4.89)	5.10 (5.05–5.15)	17.57 (17.35–17.79)
	SCC: 250,000–500,000	6.57 (6.25–6.89)	4.84 (4.77–4.91)	5.10 (5.04–5.16)	17.36 (16.99–17.73)
	SCC: 500,000–1,500,000	6.67 (6.40–6.94)	4.72 (4.64–4.80)	4.94 (4.87–5.01)	17.16 (16.87–17.43)
	SCC: 1,500,000–5,000,000	6.82 (6.47–7.17)	4.62 (4.55–4.69)	4.87 (4.79–4.95)	17.03 (16.66–17.40)
	SCC > 5,000,000	5.68 (5.21–6.15)	4.69 (4.58–4.80)	4.94 (4.79–5.09)	16.07 (15.47–16.67)
SCM ^1^ and Staphylococci Groups	SCM + CPS detected	6.29 (5.80–6.78)	4.71 (4.62–4.80)	4.96 (4.89–5.03)	16.78 (16.18–17.38)
	SCM + NSCNS detected ^3^	6.66 (6.41–6.91)	4.64 (4.55–4.73)	4.89 (4.81–4.97)	16.97 (16.69–17.25)
	SCM + NRCNS detected	6.74 (5.72–7.76)	4.71 (4.57–4.85)	4.97 (4.81–5.13)	17.25 (16.28–18.22)

^1^ According to the criteria defined in Table 1. ^2^ All examined samples were included according to their SCC, regardless of their bacteriological status. ^3^ Including two cases of mixed infections (*S. chromogenes* + *S. capitis*, *S. caprae* + *E. coli*).

## Data Availability

The original contributions presented in the study are included in the article/Supplementary Materials; further inquiries can be directed to the corresponding author.

## Supplementary Materials

The following supporting information can be downloaded at: https://www.mdpi.com/article/10.3390/vetsci11120676/s1, Table S1: Somatic cell count, and mean temperature data of healthy, unilateral SCM and bilateral SCM group specimens included in the study.
